# Solitary tracheal B-cell lymphoma in an adult alpaca (*Vicugna pacos*)

**DOI:** 10.1186/s12917-020-02640-9

**Published:** 2020-11-09

**Authors:** Emma Marchionatti, Elke Van der Vekens, Laureen Michèle Peters, Taina Susanna Kaiponen, Inês Berenguer Veiga, Patrik Zanolari

**Affiliations:** 1 Clinic for Ruminants, Department of Clinical Veterinary Medicine, Vetsuisse Faculty, University of Bern, Bremgartenstrasse 109A, 3012 Bern, Switzerland; 2 Clinical Radiology, Department of Clinical Veterinary Medicine, Vetsuisse Faculty, University of Bern, Länggassstrasse 124, 3012 Bern, Switzerland; 3 Clinical Laboratory, Department of Clinical Veterinary Medicine, Vetsuisse Faculty, University of Bern, Länggassstrasse 124, 3012 Bern, Switzerland; 4 Institute for Veterinary Pathology, Vetsuisse Faculty, University of Bern, Länggassstrasse 122, 3012 Bern, Switzerland

**Keywords:** South American Camelids, Neoplasia, Tracheal lymphoma, Ultrasound, Radiographs, Computed tomography, Immunohistochemistry

## Abstract

**Background:**

This report describes a case of solitary tracheal lymphoma in a 14-year-old alpaca mare.

**Case presentation:**

The alpaca was referred for dyspnea and inspiratory noise. The clinical examination included complete blood cell count, blood chemistry, endoscopy, ultrasound, radiographs, and computed tomography (CT). A solitary tracheal intraluminal and juxtatracheal lymphoma was diagnosed by fine needle aspiration (FNA). The owner requested euthanasia due to the uncertain prognosis. At postmortem examination, the presence of solitary lymphoma without involvement of other organs was confirmed. Immunohistochemical analysis confirmed a B-cell origin.

**Conclusions:**

Although multicentric lymphoma is the most commonly described neoplasia affecting South American camelids (SAC), solitary forms of the disease may occur.

## Background

There are occasional reports of neoplasia in South American camelids (SAC), with multicentric lymphoma being the most commonly reported neoplasm described in llamas and alpacas [1–10]. These are more often described in young alpacas (2 years of age or less) and in adult llamas (5–7 years) [3–6, 8], and the most frequently involved organs in both species include the liver, spleen, lymph nodes, kidneys and lungs [3, 4, 6, 8–10]. This report describes for the first time the case of a 14-year-old alpaca (*Vicugna pacos*) mare diagnosed with solitary tracheal B-cell lymphoma.

## Case presentation

### History

A 14-year-old, 86 kg, domestic huacaya alpaca mare, was referred to the Clinic for Ruminants, Vetsuisse-Faculty, University of Bern, Switzerland for acute dyspnea and inspiratory noise. The mare had not shown any previous respiratory symptoms and had always a good appetite. The referring veterinarian did not initiate any treatment prior to referral.

### Clinical findings

Upon clinical examination, the alpaca was bright, alert and in good general condition. Body condition was excellent (BW 86 kg; normal range 55–90 kg) with a body condition score (BCS) of 3 out of 5. Rectal temperature was 37.6 °C (range 37.5–38.9 °C), heart rate 80 beats per minute (range 60–80 bpm) and respiratory rate 28 respiration per minute (range 10–30 rpm). The mare showed mild dyspnea with bilateral dilated nostrils and inspiratory stridor. There was no nasal discharge nor spontaneous or provoked cough. Auscultation of the lung fields revealed bilateral normal sounds, whereas tracheal auscultation revealed a high-pitched stridor. Mucosal membranes were pink and moist and oxygen saturation (Datex Ohmeda Compact s/5 iMM – pulse oxymetry, GE Healthcare, USA) was 95%. Complete blood cell count and blood chemistry were performed and all values were within normal limits.

### Diagnostic imaging examinations

**Fig. 1:**
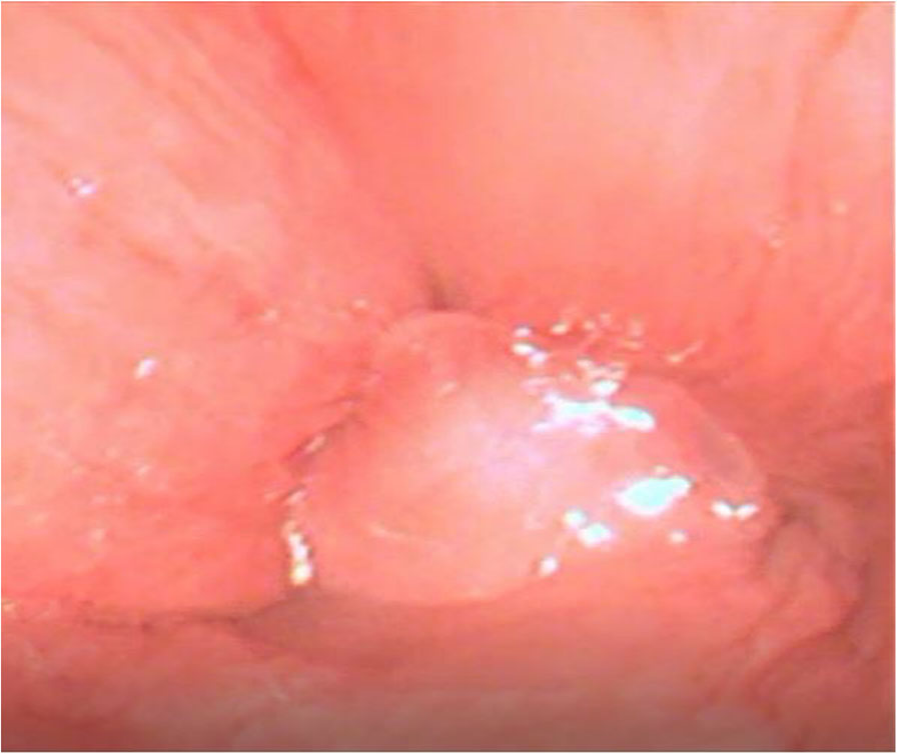
Endoscopic view of the larynx of the alpaca. Note the inability to visualize the rima glottidis and the arytenoid cartilages due to the retroversion of the epiglottis

Resting endoscopic examination (Silver Scope®, Karl Storz Endoskope, Germany; sheath 7.9 mm, length 140 cm) of the upper airway revealed mild epiglottitis characterized by mucosal edema and reddening, and epiglottic retroversion where the epiglottis retroverted in the opening of the glottis during inspiration and returned to its normal position with each expiration (Fig. 1). Nasal passages and pharynx were considered within normal limits. The trachea could not be examined endoscopically due to the epiglottic retroversion.

**Fig. 2:**
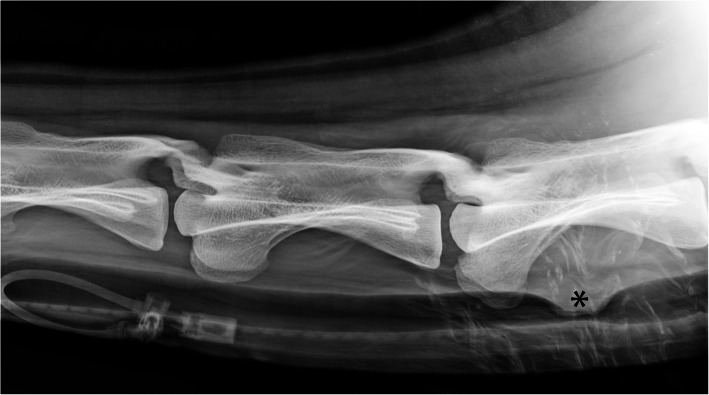
Right-to-left lateral radiograph of the neck including C3 to C5. A wide-based soft tissue opacity (*) is originating from the dorsal tracheal wall and markedly decreasing the height of the tracheal lumen at the level of C5. Note multiple wet-hair artifacts are superimposed on the ventral soft tissues at the level of C5. An IV catheter is present more cranially

Right-to-left lateral radiographic examination (Vertix Vet X-ray system, Siemens, Germany; CR-IR 342, Fuji Photo Film, Japan with FCR Fuji IP Cassette type CC 35.4 × 43 cm; standing animal with slightly extended neck) of the neck showed a wide-based soft tissue mass in the dorsal tracheal wall at the level of the 5th cervical vertebra (C5) (Fig. 2). It markedly decreased the height of the air-filled tracheal lumen and mildly deviated the cervical fascies dorsally. A mild dorsal deviation of the ventral tracheal wall was visible at the same level. The ventrodorsal projection did not provide additional information.

**Fig. 3:**
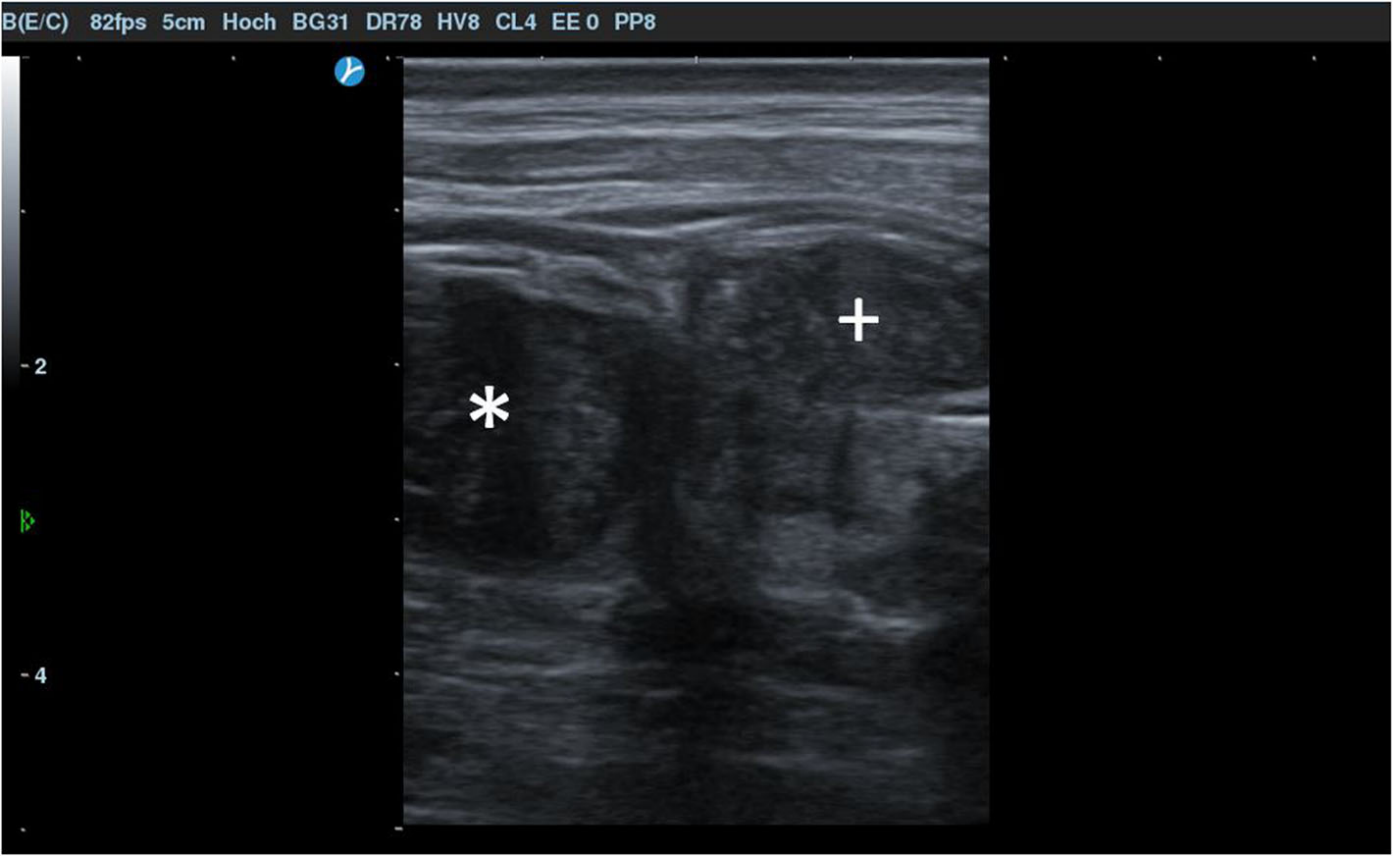
Ultrasound image of the distal third of the left aspect of the neck. An intraluminal tracheal (*) and left juxtatracheal (+) homogeneous hypoechoic masses are visible. Cranial is to the left

Ultrasound examination (Selfius UF-890AG, Fukuda Denshi, Japan; 7.5 MHz linear probe) revealed the presence of intraluminal tracheal and left juxtatracheal homogeneous hypoechoic masses at the level of the caudal third of the neck (Fig. 3). The extraluminal tracheal mass was in contact with the esophagus. Nevertheless, the motility of this latter was considered normal and not affected by possible adhesions with the mass.

**Fig. 4:**
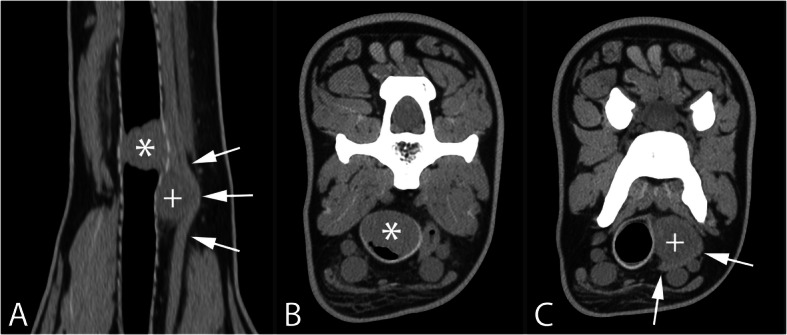
Computer tomographic dorsal **a** and transverse **b** & **c** multiplanar reconstructions of the neck using a soft tissue algorithm. A soft tissue attenuating intraluminal tracheal mass (*, **a** & **b**) is visible ventral to C4. An extra luminal soft tissue attenuating mass (+, **a** & **c**) is visible left to the trachea, displacing the esophagus (arrows) laterally and decreasing the width of the trachea

The diagnosis of a tracheal mass was made and a computer tomographic (CT) examination (Brilliance 16, Philips, The Netherlands) was performed under general anesthesia to evaluate the tracheal mass and the surrounding soft tissues in more detail. The CT examination confirmed the presence of an ovoid homogeneous soft tissue mass (LWH: 25.5 × 23 × 14.5 mm), originating from the left dorsal tracheal wall and maximally occluding +/− 90% tracheal lumen at the caudal aspect of the 4th cervical vertebra (C4) (Fig. 4a & b). The inner tracheal lining was separated from the tracheal rings cranial and caudal to this mass. Left to the trachea and dorsal to the esophagus was a second large mildly heterogeneous soft tissue attenuating mass (LWH: 21.5 × 17.5 × 20.5 mm), which focally indented the left tracheal wall and displaced the esophagus ventrolaterally (Fig. 4a & c). This second mass showed border effacement with the trachea and esophagus, but no direct connection between both masses was observed. A small soft tissue nodule (< 1 cm) was identified left ventral to the trachea, approximately 4 cm cranial to the punctum maximum of the intraluminal tracheal mass, likely representing a cervical lymph node. The left superficial cervical lymph node was moderately enlarged (33 × 9 × 2 mm), but still elongated (short axis diameter to long axis diameter ratio = 0.27), flat and with a clearly visible hilus; there were multiple mineralizations present in the normal sized contralateral lymph node (12 × 5 × 4 mm). The submandibular and medial retropharyngeal lymph nodes were symmetrical and within normal size limits.

### Cytological findings

**Fig. 5:**
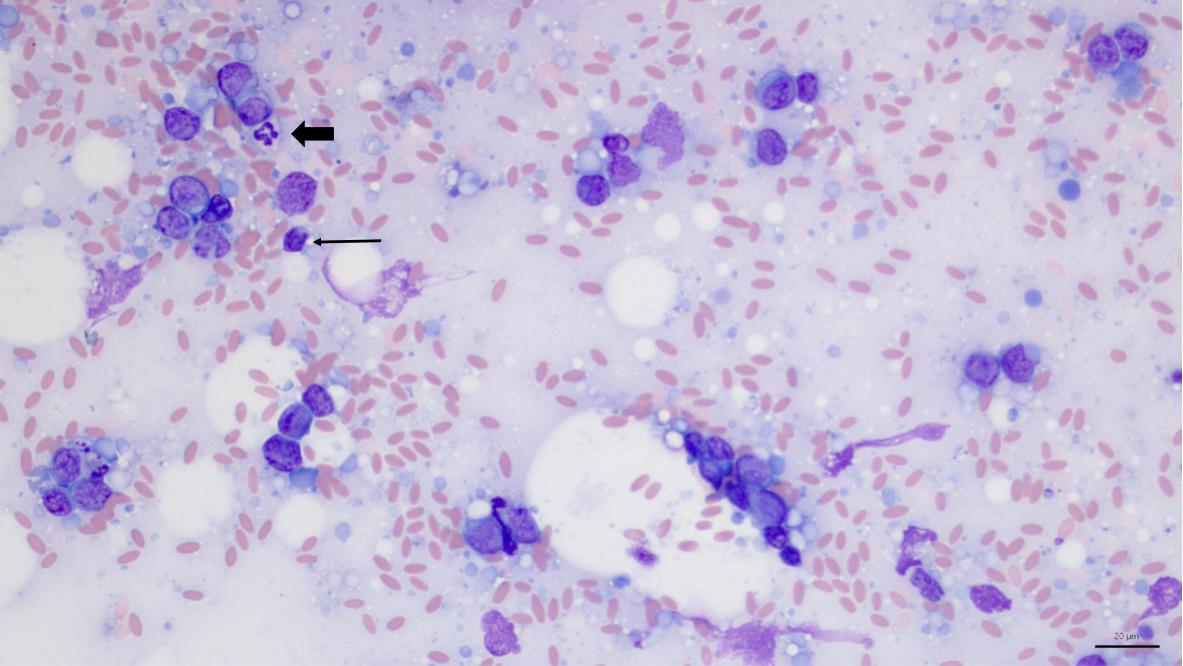
Fine needle aspirate smear. Neoplastic cells are medium sized lymphocytes, on a basophilic background with numerous cytoplasmic fragments and moderate numbers of erythrocytes. Rare small lymphocytes (thin arrow) and neutrophils (thick arrow) are highlighted for size comparison. Modified Wright’s stain, 40x objective, bar 20 μm

Based on physical examination and diagnostic imaging the main differential diagnosis was neoplastic disease. However, a granuloma could not be excluded. A fine needle aspiration (FNA) of the extraluminal mass was therefore performed under ultrasound guidance. Smears were moderately cellular and contained predominantly medium to occasionally large lymphocytes, with a round to mildly indented nucleus (approximately 10 to 15 μm in diameter), coarsely stippled chromatin, occasionally 1–2 small, variably prominent nucleoli, and low to moderate amounts of mid to deeply basophilic cytoplasm (Fig. 5). Scattered mitotic figures were observed.

A diagnosis of lymphoma was therefore issued. Due to the poor prognosis, the owner requested euthanasia of the animal. The animal was euthanized with an intravenous injection of pentobarbital (150 mg/kg IV).

### Necropsy findings

**Fig. 6:**
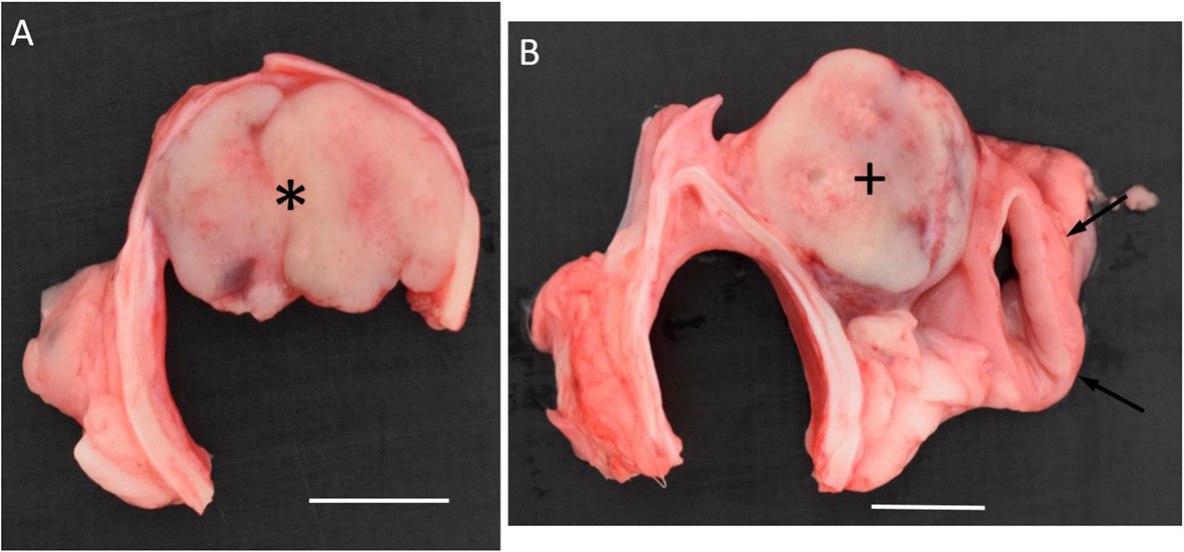
Pathology transverse sections of the trachea at the level of the intraluminal mass **a** and of the trachea and esophagus at the level of the extra luminal mass **b**. Note the subepithelial origin of the intraluminal mass (*) from the dorsal wall of the trachea and the absence of adhesions of the extra luminal mass (+) to the esophagus (arrows). White bars represent 1 cm

The postmortem examination revealed a 2 cm in diameter light pink, round, firm mass firmly attached to the left dorsal tracheal serosa, approximately 30 cm caudal to the larynx (Fig. 6b). Immediately cranial, in the lumen of the trachea, another 2 cm in diameter mass was firmly attached to the mucosa of the dorsal wall of the trachea and displayed a similar morphology as the previously described (Fig. 6a). The esophagus was displaced to the left at the level of the extra luminal mass, but no adhesions were found (Fig. 6b). With the exception of the presence of few mineralized nodules in the liver, the remaining internal organs including the lung and the cervical lymph nodes were macroscopically unremarkable.

### Histopathological and immunohistochemical findings (Fig. 7)

Samples of the tracheal masses and from the main internal organs were fixed in 10% neutral buffered formalin for histological examination. The samples were routinely processed, paraffin wax embedded, stained with hematoxylin and eosin (H&E), cut in 3 μm sections and observed in standard light microscopy.

**Fig. 7:**
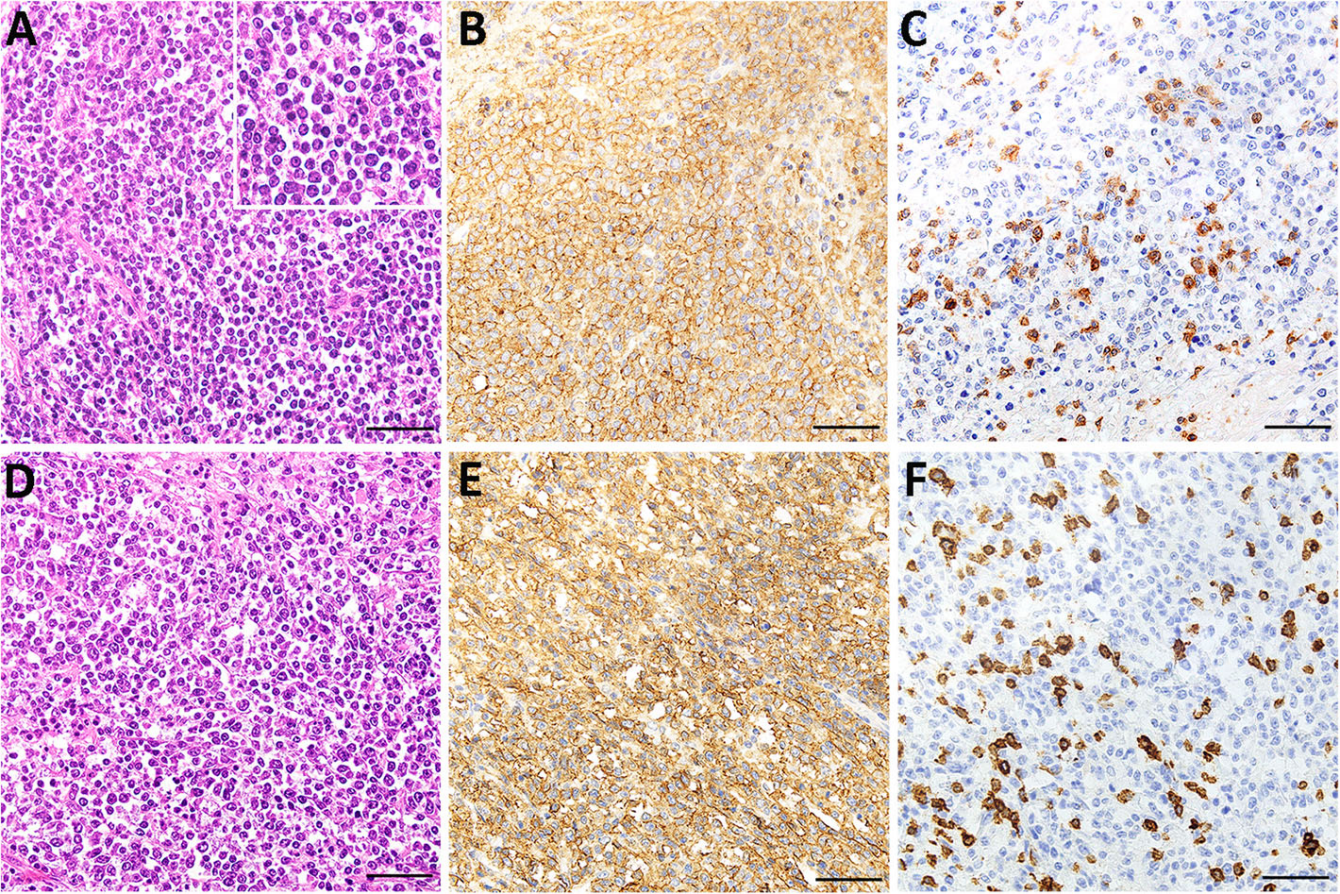
Histological and immunohistochemical analysis of the tracheal masses. **a** and **d**) Both the intraluminal **a** and extraluminal **d** masses consisted of sheets of neoplastic lymphocytes growing on a scant fibrovascular stroma. Hematoxylin and eosin (H&E), bar 50 μm. (inset A) The neoplastic lymphocytes displayed a small amount of eosinophilic, homogeneous cytoplasm, and a round nucleus with finely stippled chromatin. **b** and **e** The neoplastic lymphocytes observed in the intraluminal **b** and extraluminal **e** masses displayed a strong CD20 membranous signal. CD20 immunohistochemical staining, bar 50 μm. **c** and **f** Occasional CD3 positive lymphocytes, which most likely represent reactive non neoplastic cells, could be observed among the neoplastic lymphocytes in both the intraluminal **c** and extraluminal **f** masses. CD3 immunohistochemical staining, bar 50 μm

Histologically, the intraluminal tracheal mass consisted of a highly cellular, well demarcated, unencapsulated and infiltrative growing proliferation of neoplastic round cells within the lamina propria, which obstructed the tracheal lumen almost completely and whose surface was focal- extensive ulcerated. The neoplastic cells grew in sheets on a scant amount of highly vascularized fibrous stroma, displayed a small amount of eosinophilic, homogeneous cytoplasm, a round nucleus measuring 1–2 times the length of an erythrocyte with finely stippled chromatin and up to 4 round, basophilic nucleoli (Fig. 7a). The anisocytosis and the anisokaryosis were moderate to high, and there were 14 mitotic figures in ten 400x high-power-fields. In addition, the mass displayed extensive necrotic areas, as well as a multifocal, moderate infiltration with non-neoplastic lymphocytes. The extraluminal tracheal mass displayed similar histological characteristics as the intraluminal mass (Fig. 7d). In addition, a mild suppurative bronchopneumonia, which was most likely due to an impairment of the mucociliary apparatus of the conducting system due to the presence of the intraluminal tracheal mass, and a mild, multifocal chronic granulomatous and fibrosing cholangiohepatitis with multifocal mineralization, which was most likely due to a parasitic infection, could be detected histologically. No further pathological changes were observed histologically in the remaining analyzed internal organs.

Immunohistochemical stains for CD20 (1:200 dilution of Lab Vision™ anti-CD20 rabbit polyclonal antibody, Thermo Fisher Scientific, USA) and CD3 (1:100 dilution of clone LN10 anti-CD3 antibody, Leica Biosystems, Germany) were then performed in order to further characterize these neoplastic lymphocytes. The spleen and a mediastinal lymph node from an alpaca that died due to cachexia and tissue sections from the intraluminal tracheal mass without primary antibody addition were used as positive and negative controls, respectively. The neoplastic lymphocytes in both masses showed a strong positive membranous staining in the CD20 stain (Fig. 7b and e). Occasional CD3 positive lymphocytes displaying both membranous and cytoplasmic staining (Fig. 7c and f), which most likely corresponded to the reactive lymphocytic infiltrates observed in the H&E staining, were also observed. Diagnosis of a B-cell tracheal lymphoma was therefore confirmed.

## Discussion and conclusions

Although multicentric lymphoma is the most commonly reported neoplasm described in SAC [1–10], this particular case is, to our knowledge, the first report of a solitary tracheal B-cell lymphoma in a geriatric alpaca. Underlying factors that could predispose to lymphoma development in SAC haven’t been identified to date, even if a bovine leukemia virus induced lymphoma has been previously described in a 13-month-old alpaca [11]. Young alpacas (up to 2 years old) of both genders, may develop a disseminated form resembling juvenile lymphoma in calves [6, 8, 10], while adult llamas (5–7 years) also seem to be particularly predisposed to lymphoma development [3–6, 8].

Immunohistochemical staining using CD3, PAX-5, BLA36, CD 79 and CD20 [8, 10, 12, 13] allows the differentiation of these tumors into T- or B-cell lymphomas. Although B-cell lymphomas appear to occur more frequently in SAC [5, 6, 8], the juvenile disseminated form observed in young alpacas is usually of T-cell origin [10]. Primitive malignant round cell tumors (PMRCT) that stain with neural markers, but not with T- or B-cell markers correspond to approximately 25% of the round cell tumors detected in SAC [8].

Primary tracheal neoplasia are uncommon in animals and humans. In humans, tracheal neoplasia account for up to 0.2% of all tumors, with most being squamous cell carcinoma or adenoid cystic carcinoma [14, 15]. Lymphoma accounts for only 0.2–3% of tracheal neoplasia in humans [16–18] and is only very seldom described in veterinary medicine [19, 20]. A tracheal B-cell lymphoma was previously reported in a 3-year-old llama. However, since this animal displayed also neoplastic changes in the kidney, it is unclear whether this neoplasia was primarly intratracheal [10].

In the present case, it could not be determined with certainty whether this neoplasia had an intraluminal or extraluminal tracheal origin. However, since both masses displayed similar histopathological and immunohistochemical characteristics, a common origin is assumed. In human medicine, intraluminal tracheal B-cell lymphomas have often been characterized as mucosa associated lymphoid tissue (MALT) lymphomas. These are low-grade malignant neoplasias, which often remain localized and are generally associated with a favorable outcome [21, 22]. In our case, the presence of the extraluminal tracheal mass adjacent to the intraluminal mass suggests a local invasive behavior (for example of a deep cervical lymph node), but there was no clear histological evidence of vascular or lymphatic invasion, and no further metastases could be detected.

Thoracic radiography is the initial imaging study being performed in humans presenting signs of dyspnea, wheezing and/or hemoptysis, suggestive of a tracheal neoplasia. This imaging modality, however, identifies only 18–28% of tracheal tumors [15]. Neck radiography was successful, in the present case, in allowing detection of a soft tissue opacity causing a severe narrowing of the tracheal lumen. Bronchoscopy and CT represent, however, the gold standard for diagnosis of tracheal neoplasms in humans [15, 23]. Bronchoscopy allows direct visualization and evaluation of mucosal lesions in the airway lumen, as well as possibility to obtain tissue samples, but determination of disease extension is difficult. Computed tomography is the imaging modality of choice for determination of location and size of intraluminal neoplasms, extra luminal extension of the disease and invasion of adjacent structures, and presurgical planning. Endoscopic evaluation of the lymphoma described in the present report was not possible due to the epiglottic retroversion. This condition could be explained by the increased turbulent airflow due to the extremely severe reduction in the tracheal lumen and the consequent increased respiratory effort producing a suction effect during inspiration. The CT-scan was helpful, in the present case, to provide a better overview of the condition of the trachea and adjacent structures. However, the classification of the lesion remained difficult and a FNA was necessary to provide a conclusive diagnosis in the living animal.

Treatment strategies in human medicine include surgical excision, chemotherapy and radiation therapy, alone or in combination. Reports of chemotherapy in SAC are rare. Cebra et al. reported an attempt using a combination of cyclophosphamide, vincristine and prednisolone for treatment of a llama diagnosed with a multicentric lymphoma, but with only a transient and short-duration response [3]. Radiation therapy has been reported in SAC as adjuvant treatment for fibrosarcoma, sarcoma and ameloblastoma, but with inconsistent results [24–26]. In the present case, the owner elected euthanasia of the animal due to the uncertain prognosis and unpredictable effect of chemo- and radiotherapy in SAC.

This report describes the clinical, diagnostic imaging, and pathological findings of a solitary tracheal B-cell lymphoma in a geriatric alpaca mare. As SAC are constantly increasing in popularity as pets, information regarding uncommon diseases become important for the veterinarian. Although multicentric lymphoma is the most common neoplasm affecting SAC, solitary forms of the disease may occur. Further studies are needed regarding the therapeutic options of neoplasms in SAC.

## Data Availability

The patient data used in this case report are available from the corresponding author on reasonable request.

## Abbreviations

SAC: South American camelids
BW: Body weight
BCS: Body condition score
FNA: Fine needle aspirate
CT: Computed tomography
MALT: Mucosa associated lymphoid tissue
